# New advances in CRISPR/Cas-mediated precise gene-editing techniques

**DOI:** 10.1242/dmm.049874

**Published:** 2023-02-27

**Authors:** Chris Richardson, Robert N. Kelsh, Rebecca J. Richardson

**Affiliations:** ^1^ School of Physiology, Pharmacology and Neuroscience, Faculty of Biomedical Sciences, University of Bristol, Bristol BS8 1TD, UK; ^2^ Department of Life Sciences, University of Bath, Bath BA2 7AY, UK

**Keywords:** CRISPR/Cas, HDR, Precise genome editing, Base/prime editing, Human disease modelling

## Abstract

Over the past decade, CRISPR/Cas-based gene editing has become a powerful tool for generating mutations in a variety of model organisms, from *Escherichia coli* to zebrafish, rodents and large mammals. CRISPR/Cas-based gene editing effectively generates insertions or deletions (indels), which allow for rapid gene disruption. However, a large proportion of human genetic diseases are caused by single-base-pair substitutions, which result in more subtle alterations to protein function, and which require more complex and precise editing to recreate in model systems. Precise genome editing (PGE) methods, however, typically have efficiencies of less than a tenth of those that generate less-specific indels, and so there has been a great deal of effort to improve PGE efficiency. Such optimisations include optimal guide RNA and mutation-bearing donor DNA template design, modulation of DNA repair pathways that underpin how edits result from Cas-induced cuts, and the development of Cas9 fusion proteins that introduce edits via alternative mechanisms. In this Review, we provide an overview of the recent progress in optimising PGE methods and their potential for generating models of human genetic disease.

## Teaser

**Summary:** CRISPR/Cas technology can effectively disrupt gene function by generating insertions and deletions, but precise gene editing remains challenging. Here, we discuss recent advances in this field.

## Introduction

**Fig. 1.**
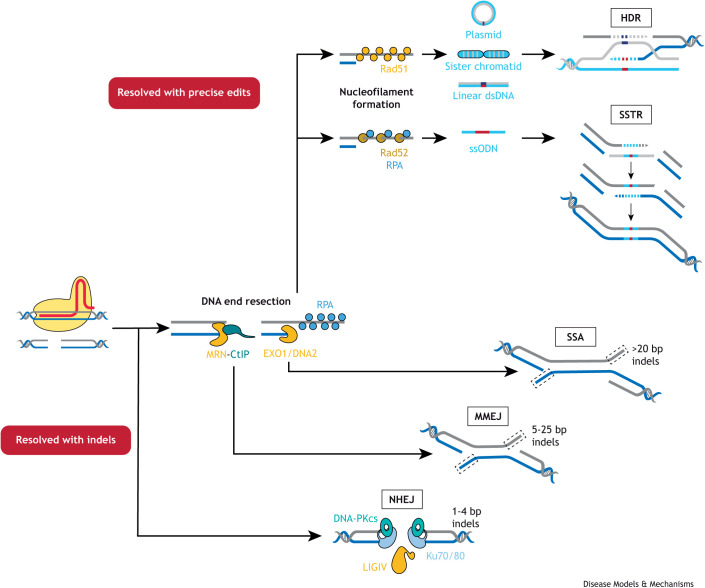
**Summary of known double-stranded break (DSB) repair pathways.** After initiation of a DSB, the initial repair pathway decision is between the binding of the dsDNA ends with Ku70/80 and subsequent error-prone NHEJ (bottom) or DNA end resection by the MRN complex, which is activated by CtIP (middle). Before extensive DNA end resection occurs, the micro-homologies between the exposed ssDNA ends can facilitate a second alternative error-prone repair mechanism, MMEJ. Alternatively, more extensive DNA end resection is carried out by EXO1 and DNA2, after which the exposed ssDNA is bound by RPA. This initiates one of two faithful repair mechanisms (top). When Rad51 is successfully loaded onto the ssDNA strand and a nucleofilament is therefore formed, the DSB can be resolved via HDR in the presence of a dsDNA donor. Alternatively, two other DSB resolution fates can occur: homologies within the exposed ssDNA may facilitate the third error-prone repair mechanism, SSA, or Rad52 may be loaded onto the ssDNA and can trigger the faithful repair mechanism, SSTR, if a ssODN template is present at the site of the DSB. Importantly, NHEJ, the main competitor of faithful repair mechanisms, can readily occur at the earliest stage in the DSB resolution process, with subsequent error-prone repair mechanisms still occurring either part way through and/or after extensive DNA end resection. By contrast, both known faithful repair mechanisms require extensive DNA end resection and can be subdivided into Rad51-dependent HDR and Rad51-independent SSTR. dsDNA, double-stranded DNA; HDR, homology-directed repair; indel, insertion or deletion; MMEJ, microhomology-mediated end joining; MRN, MRE11, Rad50 and NSB1; NHEJ, non-homologous end joining; SSA, single-strand annealing; ssODN, single-stranded oligodeoxynucleotide; SSTR, single-stranded template repair.

Gene editing via CRISPR/Cas technology has become a key tool for researchers in many areas, including plant and agricultural biology, human disease and synthetic biology. The relative ease of its implementation in many systems, availability of bioinformatic tools (Heigwer et al., 2014; Labun et al., 2019; Liu et al., 2015), commercially available reagents, flexibility and effectiveness all combine to make CRISPR/Cas an invaluable tool for modifying gene and protein function *in vitro* and *in vivo*. The *Streptococcus pyogenes* Cas9 (SpCas9) endonuclease has become a workhorse for generating genetic knockouts, as well as facilitating more precise edits (Anders et al., 2014; Jinek et al., 2012). Cas9-based gene-editing approaches exploit two key features of this endonuclease: its ability to recognise and bind to specific DNA sequences based on the complementarity of a guide RNA (gRNA) (Anders et al., 2014), and its ability to introduce double-stranded breaks (DSBs) in the target DNA strand (Jinek et al., 2012) (Fig. 1). Cas9 gRNAs are composed of two small non-coding RNAs: a uniform trans-activating CRISPR RNA (tracrRNA) that is recognised by the Cas9 and a CRISPR RNA (crRNA) that contains the locus-specific sequence. Together, these form a duplex called a single-guide RNA (sgRNA), which then forms a ribonucleoprotein (RNP) complex with Cas9 (Karvelis et al., 2013). Other Cas endonucleases, such as Cas12a, only require crRNAs (Zetsche et al., 2015; Zetsche et al., 2017). To cover all types of Cas endonucleases, we use the collective term ‘gRNA’ in this Review. gRNAs require the presence of a protospacer adjacent motif (PAM) (Anders et al., 2014), a specific sequence of nucleotides without which Cas will not cut. PAM site specificity, such as NGG for SpCas9, however, limits the loci that Cas can be targeted to.

The ability of Cas endonucleases to generate DSBs triggers the recruitment of the endogenous DNA repair machinery to the break, and a specific genetic locus can be targeted via a gRNA. The most common DNA repair pathway, non-homologous end joining (NHEJ), results in the introduction of insertions or deletions (indels) due to the error-prone nature of this repair mechanism (Fig. 1). However, the initiation of DSBs also allows for the possibility of faithful homologous recombination (HR)-driven repair mechanisms, such as homology-directed repair (HDR) or single-stranded template repair (SSTR), which utilise a donor sequence to repair the DSB, allowing the engineered incorporation of specific edits into the target strand (Fig. 1). Although NHEJ is active throughout the cell cycle, HDR and SSTR are limited to the S and G2 phases, when sister chromatids would naturally be located close together to act as a repair template (Heyer et al., 2010).

Gene knockout or mutant disease models have been generated using numerous techniques, including chemical mutagenesis, such as with N-ethyl-N-nitrosourea (ENU) (de Angelis et al., 2000; Fossett et al., 1990; Hitotsumachi et al., 1985; Solnica-Krezel et al., 1994), engineered endonucleases such as transcription activator-like effector nucleases (TALENs) (Ke et al., 2016; Li et al., 2011), targeted genetic modification of mammalian embryonic stem cells to generate chimeric modified mice (Mak, 2007) or rescue of knockout models with humanised sequences containing disease-associated mutations (Switonski et al., 2015). However, these techniques can be laborious, chemical mutagenesis cannot be targeted to a specific region of the genome, and rescue with humanised mutation-bearing constructs is not always faithful to the expression level of the endogenous gene. More recently, the use of Cas endonucleases to generate indels has been optimised in many systems: efficiencies can exceed 90% *in vivo* in zebrafish (Kroll et al., 2021; Burger et al., 2016), with recent adaptions for high-throughput screening (Parvez et al., 2021), 85% in mammalian cell culture (Seki and Rutz, 2018), 66% in *Arabidopsis thaliana* (Tsutsui and Higashiyama, 2016) and 85.7% in rats (Bae et al., 2020). Indel formation, arising as a result of DSB repair via NHEJ and leading to loss-of-function gene disruption, can be used to study gene and protein function and can model many diseases (Hai et al., 2014; Kang et al., 2015; Yuan et al., 2016; Wang et al., 2019; Stenson et al., 2003). However, in many cases, diseases are caused by single-nucleotide substitutions rather than full gene disruption (Stenson et al., 2003), and the resulting subtle changes in function cannot always be fully replicated with knockout models (Hancox et al., 2019; De Gobbi et al., 2006; Ingram, 1957). Therefore, generation of targeted, specific mutations in endogenous genes is desirable.

Modelling such genetic alterations requires precise genome editing (PGE) methods. However, PGE methods that rely on HDR/SSTR to resolve Cas-induced DSBs are significantly less efficient than NHEJ-driven indel formation. PGE methods often achieve <4% efficiency (Cui et al., 2018; Prykhozhij et al., 2017 preprint; Wu et al., 2013) (Table 1), making the generation of precise human disease models, particularly *in vivo* (Box 1), laborious and expensive. Therefore, optimisations to PGE methods and efficiencies are urgently required. Increasing PGE efficiency is a multifaceted challenge that will likely require a combination of approaches to solve. These include efficient DNA cleavage, efficient delivery of PGE components into the target cell, the timing of editing, careful design and choice of donor DNA, the ability to effectively pass on mutations to the next generation in model organisms and the capability to subsequently screen for desired mutations. Importantly, these also require optimisation of strategies to manipulate the competitive DSB repair pathways that can either give rise to indels or PGE. Although robustly and reproducibly increasing PGE efficiency remains a challenge, there have been considerable recent advances, which we summarise in this Review. We discuss recent progress in multiple model systems and provide an overview of methods that can be employed to increase PGE efficiency.

**Box 1. Use of precise genome editing (PGE) *in vivo* for the generation of disease models**

Many developments in CRISPR technology are first investigated and proven *in vitro* (Table 1) as these platforms allow researchers to elucidate how the genome-editing process and the resulting mutations affect protein and cell function. Translation of these methods to generate *in vivo* human disease models is a powerful use of this technology. However, adapting methods developed *in vitro* for use *in vivo* is challenging. In cell culture, thousands of cells can be transfected with PGE components simultaneously, whereas germ-line mutations in whole animals typically require skilled and time-consuming manual microinjection of PGE components into fertilised embryos (the F0 generation) (Cui et al., 2018; Lamas-Toranzo et al., 2020; Park et al., 2017; Zhang et al., 2018; Song et al., 2018; Tessadori et al., 2018; Aksoy et al., 2019). Furthermore, the end goal of *in vivo* modelling is typically a stable precisely edited strain, which also requires efficient transmission of the edits to offspring (the F1 generation).

Despite these challenges, PGE has been achieved in several model organisms, including mice (Miura et al., 2018; Wu et al., 2013), zebrafish (Hoshijima et al., 2016; Irion et al., 2014; Wierson et al., 2020; Zhang et al., 2018) rabbits (Song et al., 2016; Song et al., 2018), pigs (Song et al., 2016; Song et al., 2018) and non-human primates (Lamas-Toranzo et al., 2020; Wang et al., 2016; Yan et al., 2018). The efficiency of *in vivo* PGE methods, however, varies considerably between experiment, model organism and targeted loci (Table 1). For example, *in vitro* use of the DNA-PKcs inhibitor NU7441, which blocks non-homologous end joining and thus promotes PGE repair pathways, increased PGE efficiency to between 6.2% and 15% in HEK293 cells and induced pluripotent stem cells (Cui et al., 2018; Kang et al., 2019). When used *in vivo*, in zebrafish, NU7441 addition resulted in 50% somatic PGE and three of six fish produced chimeric-edited F1 progeny (Aksoy et al., 2019). Conversely, using single-stranded oligodeoxynucleotides (ssODNs) and foregoing chemical modulation for PGE in pigs resulted in between 0% and 60% of viable F0 piglets being mosaic for the desired edits and this varied between litters/experiments (Park et al., 2017). These differences in PGE efficiencies make it difficult to compare methods between studies and underscore the broad variability between organisms and protocols.

Although efficient *in vivo* gene editing remains challenging, human diseases caused by single-nucleotide substitutions have been successfully modelled *in vivo* using PGE methods. Base editing has been employed in zebrafish to generate cancer models by precise mutation of *tp53* and *nras* (Rosello et al., 2021). ssODN donor templates have been used to generate gene-edited mice that carry single-nucleotide polymorphisms implicated in human thrombosis and platelet function, which were identified from genome-wide association studies (Zhu et al., 2017). A linearised plasmid has also been used to generate knock-in pig models of Huntington's disease that exhibited germ-line transmission of the edited alleles to F1 and F2 generations (Yan et al., 2018). Such examples show the potential of Cas-based PGE for producing animal models for human genetic diseases, which can be used to better understand the function of disease-associated mutations and develop more personalised treatments.

**Table 1.**
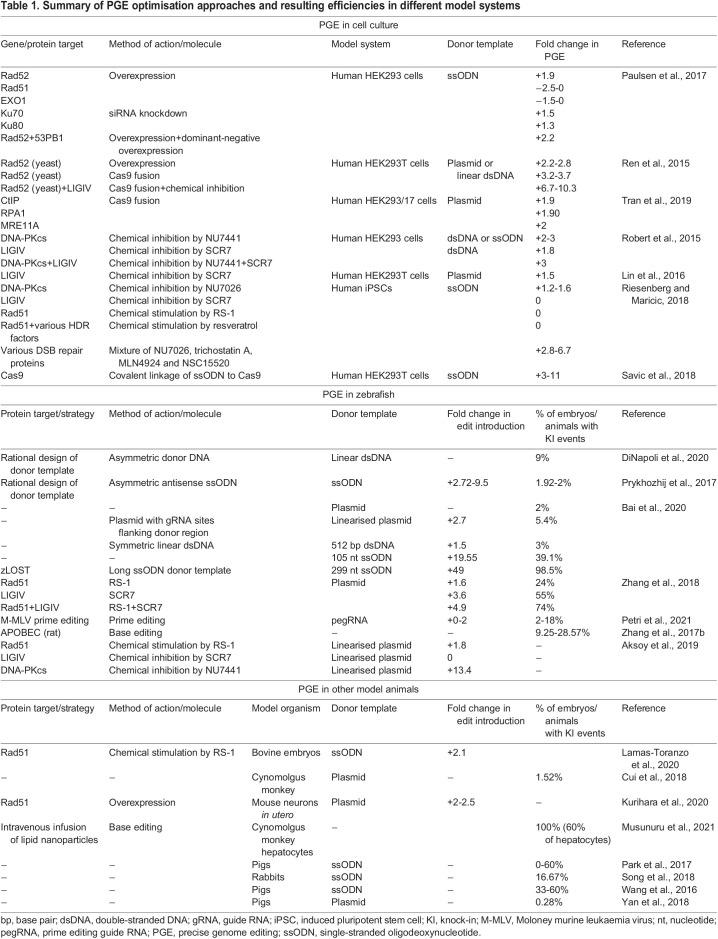
Summary of PGE optimisation approaches and resulting efficiencies in different model systems

## The use of the CRISPR/Cas system for precise genome editing

The induction of DNA repair mechanisms upon Cas-induced breaks creates the opportunity to supply exogenous donor DNA (dDNA) that contains the required change of sequence. dDNA templates for HDR or SSTR are composed of homology arms flanking the desired edits. These edits can be single-base-pair changes, specific deletions or insertions ranging from a few base pairs to complete loxP sites (Yang et al., 2013), or molecular tags or fluorescent reporters (Wierson et al., 2020; Aksoy et al., 2019; Kurihara et al., 2020). When generating such precise edits, scarless integration is desired at a specific locus, driven by the specificity of the gRNA, with the flanking recipient genomic DNA sequence remaining unaltered. Such an event, regardless of method, is encapsulated by the term PGE. Different methods exist within this umbrella term, separated by their mode of action: HDR and SSTR are separate DNA repair mechanisms that can be harnessed using dDNA (Fig. 1), whereas the more recently described base editing (BE) and prime editing (PE) employ different mechanisms to alter the target DNA sequence, which we discuss in more detail below.

Researchers have used various strategies to tackle PGE optimisation and each will be discussed in this Review. Some studies have focused on altering which DSB repair pathway is favoured, either through chemical modulation (Aksoy et al., 2019; Bischoff et al., 2020; Zhang et al., 2018) or through overexpression or silencing of key genes in the HDR/SSTR or NHEJ pathways (Kurihara et al., 2020; Li et al., 2018). The search for alternative Cas endonucleases (Makarova et al., 2011) has expanded the genome-editing toolbox to include Cas12a (Cpf1) and variants of Cas9, such as *Staphylococcus aureus* Cas9 (SaCas9) (Kleinstiver et al., 2015a; Ran et al., 2015) and *Neisseria meningitidis* Cas9 (NmCas9) (Hou et al., 2013), which have differing target preferences (Chen et al., 2018; Swarts and Jinek, 2018; Zetsche et al., 2015; Nakade et al., 2017) and thus allow the targeting of new loci. Alteration (Walton et al., 2020) of the Cas9 PAM preference (Doudna and Charpentier, 2014; Mojica et al., 2009) has likewise increased the spectrum of potential genetic targets, while identification of high-fidelity Cas9 variants has increased specificity (Walton et al., 2020; Kleinstiver et al., 2016, 2015b).

The ability of Cas to act as a genetic homing mechanism has also been exploited independently of its endonuclease activity. Cas9 variants that lack the ability to introduce DSBs are termed dead Cas9 (dCas9) if they lack any endonuclease activity or Cas9 nickase (nCas9) if they only cut one DNA strand (Cong et al., 2013). dCas9 and nCas9 have been used to reduce observed off-target effects (Ran et al., 2013; Mali et al., 2013) and as a chassis onto which to fuse other enzymes, such as deaminases (Gaudelli et al., 2017; Kim et al., 2017b; Komor et al., 2016, 2017) and reverse transcriptases (Anzalone et al., 2019; Liang et al., 2022; Lin et al., 2020; Petri et al., 2021), which can induce PGE via non-HR methods, such as BE and PE. Cas9 fusions have also been used to increase PGE efficiency by assisting with the colocalisation of the dDNA or of key PGE effector proteins to the DSB site (Charpentier et al., 2018; Savic et al., 2018) (Table 1).

Different dDNA conformations have also been investigated. Researchers have compared PGE efficiencies of circular plasmid DNA, double-stranded DNA (dsDNA), single-stranded oligodeoxynucleotides (ssODNs) and long single-stranded oligodeoxynucleotides (lssODNs) (Bai et al., 2020; Miura et al., 2018; Ranawakage et al., 2021; Song and Stieger, 2017), dDNA length, strand complementarity and symmetry. These parameters have all been reported to affect HR efficiency (Okamoto et al., 2019; Richardson et al., 2016) (Table 1). In addition, researchers have developed a number of bioinformatics tools to assist with the design of dDNA and gRNAs for optimal HDR efficiencies (O'Brien et al., 2019; Prykhozhij et al., 2021).

## Manipulation of key DSB repair proteins to alter repair pathway choice

**Fig. 2.**
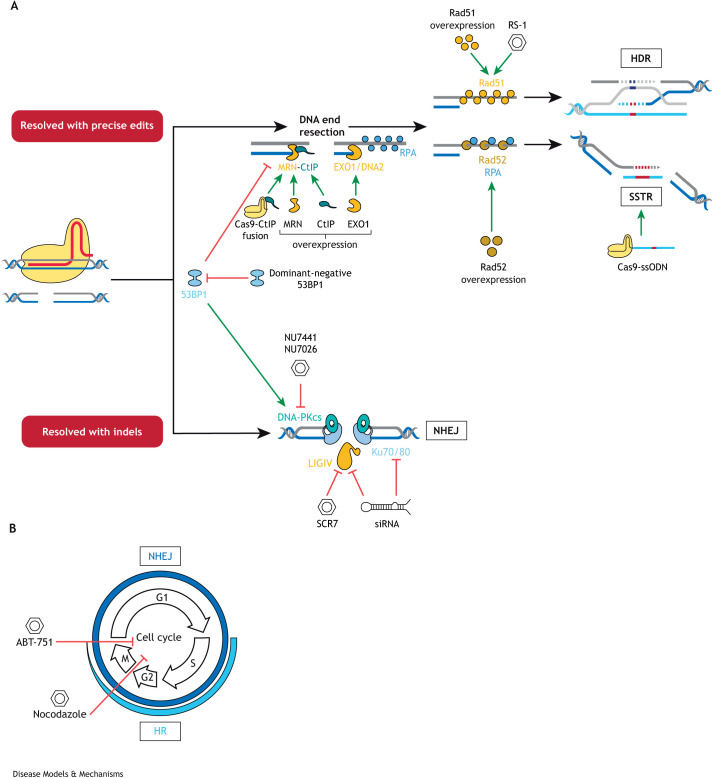
**Summary of strategies to modulate DSB repair to increase precise genome editing (PGE) efficiency.** (A) The upper track represents the desired route towards PGE, subdivided into HDR and SSTR. The lower route represents error-prone NHEJ. Several components of each pathway have been targeted for genetic or chemical inhibition and for upregulation, for example, via overexpression. These interventions skew endogenous DNA repair towards faithful mechanisms that allow PGE. (B) Modulating the cell cycle by arresting the cells in the G2/M phases can also increase PGE, as HDR and SSTR occur exclusively in those phases. HDR, homology-directed repair; HR, homologous recombination; MRN, MRE11, Rad50 and NSB1; NHEJ, non-homologous end joining; siRNA, small interfering RNA; ssODN, single-stranded oligodeoxynucleotide; SSTR, single-stranded template repair.

Processes involved in DNA repair are a necessary bedrock on which to build an understanding of PGE optimisation. In-depth descriptions of DSB repair pathways have been provided elsewhere (Shrivastav et al., 2008; Ceccaldi et al., 2016; Hustedt and Durocher, 2017) and will not be covered in detail here. Briefly, numerous proteins are involved in the repair of DSBs (outlined in Fig. 1). Six core components of NHEJ have been identified: Ku70/80, DNA-PKcs (also known as PRKDC), Artemis, LIGIV and XRCC4. Ku70 and Ku80 form a heterodimer that binds to DNA ends and recruits DNA-PKcs. DNA-PKcs is a kinase that phosphorylates Artemis, an endonuclease that trims the DSB ends and prepares them for ligation by LIGIV. Finally, XRCC4 acts as a scaffolding protein (Andres et al., 2012) and forms a complex with LIGIV (Drouet et al., 2005) to assist with its nuclear import (Berg et al., 2011). The suppression of LIGIV activity in zebrafish (Zhang et al., 2018) and silencing of Ku70/80 in pig foetal fibroblasts (Li et al., 2018) or XRCC4 in plants (*Populus trichocarpa*) (Movahedi et al., 2022) all increase PGE events, indicating that downregulation of NHEJ components is a promising avenue for increasing CRISPR-mediated PGE efficiency (Fig. 2).

An alternative approach to increasing PGE is to upregulate components of faithful repair mechanisms. As described in Fig. 1, DNA end resection is the first step towards HDR or SSTR. As such, the end resection-initiating MRN complex, formed of MRE11, Rad50 and NSB1 and activated by CtIP (also known as RBBP8), is a potential target for upregulation (Figs 1 and 2). Indeed, overexpression of CtIP and MRE11 (Movahedi et al., 2022) and fusion of a CtIP domain to Cas9 (Charpentier et al., 2018) increase PGE events. Additionally, the nucleases EXO1 and DNA2 catalyse the more extensive DNA end resection that faithful repair mechanisms require, and overexpression of an EXO1 mimic increases SSTR in mammalian cell culture (Seigel, 2018) (Fig. 2).

Once extensive DNA end resection has occurred, the resulting exposed single-stranded DNA (ssDNA) is bound by RPA (Wobbe et al., 1987; Wold, 1997), and the repair pathways available to the cell then include HDR, SSTR and single-strand annealing (SSA) (Fig. 1). The recombinases Rad51 and Rad52 appear to be key mediators of DSB resolution at this stage, but the type of template available for repair is also critical. Rad51 facilitates HDR using a dsDNA template, whereas overexpression of Rad52 has been shown to increase PGE via SSTR with ssODN templates (Gallagher and Haber, 2021; Gallagher et al., 2020). Overexpression of Rad51 increases PGE events with double-stranded plasmid dDNA *in utero* in mice (Kurihara et al., 2020), in rabbit embryos (Song et al., 2016) and in human embryonic and induced pluripotent stem cells (Takayama et al., 2017) (Fig. 2). In addition, small molecules selected for their ability to stimulate Rad51 drive similar increases in PGE in rabbit (Song et al., 2016) and zebrafish (Zhang et al., 2018) embryos. However, when using ssODN donors, which require SSTR, overexpression of Rad51 appears to be detrimental to the rate of PGE (Paulsen et al., 2017). Instead, overexpression of Rad52 increases PGE events when using single-stranded dDNA (Paulsen et al., 2017) (Fig. 2).

BRCA1 and BRCA2 also play key roles in facilitating DSB repair via HDR and SSTR (Roy et al., 2012). BRCA1 likely facilitates DNA end resection by recruiting CtIP to the DSB (Yun and Hiom, 2009; Chen et al., 2008). In a separate function, BRCA1 promotes BRCA2 localisation to resected ssDNA via the intermediary protein PALB2 (Xia et al., 2006; Zhang et al., 2009). Subsequently, BRCA2 loads Rad51 onto the RPA-coated ssDNA, enabling nucleofilament formation (Thorslund et al., 2010; Liu et al., 2010). Overexpression of BRCA1 variants that display a hyper-recombination phenotype increases PGE when using plasmid donors *in vitro* compared to wild-type BRCA1 overexpression (Pinder et al., 2015).

The examples above demonstrate a clear potential to increase the efficiency of PGE events via the manipulation of individual components of the DNA repair pathways. This approach presents a promising avenue of inquiry towards the ultimate development of super-high-efficiency Cas-mediated PGE in both *in vitro* and *in vivo* systems (Table 1). The rational step of combining simultaneous downregulation of proteins that promote error-prone repair with upregulation of proteins that mediate faithful repair has also been explored. For example, combining overexpression of Rad52 and a dominant-negative form of 53BP1 (also known as TP53BP1), a protein that supresses DNA end resection to favour NHEJ (Chapman et al., 2013), significantly increases ssODN donor-mediated PGE events in induced pluripotent stem cells (Paulsen et al., 2017). In a similar approach, the small molecules RS-1 and SCR7, which are proposed to upregulate Rad51 and downregulate LIGIV activity, respectively, significantly increase the frequency of PGE events in zebrafish embryos (Zhang et al., 2018).

## Chemical modulation of PGE

As exemplified in the study summarised above (Zhang et al., 2018), small molecules are an attractive option for the modulation of DNA repair pathways and optimisation of PGE. One benefit is that they can interact directly with proteins that already exist within the target cell, unlike small interfering RNAs (siRNAs) or morpholinos that target mRNA transcripts. This direct function facilitates more rapid effects. From a practical standpoint, it may also be easier to dissolve pre-synthesised chemicals in cell culture or embryo medium for delivery into the cells or tissue, rather than producing and delivering RNA for overexpression or knockdown of endogenous transcripts. For these reasons, researchers have investigated a range of chemicals that affect DNA repair proteins. Although promising, chemical interventions using small molecules to alter the activity of DSB repair proteins have had somewhat variable success (Table 1). For example, RS-1 and SCR7 were effective in some cases yet had small or negative effects on PGE efficiency in others (Zhang et al., 2018; Riesenberg and Maricic, 2018). The specific interplays between chemicals and the dDNA type are a potential explanation for this variability, especially when considering the Rad51-dependent nature of HDR compared to Rad52-mediated SSTR. The proposed effect of RS-1 is to stabilise the binding of Rad51 to the resected target strand (Jayathilaka et al., 2008). Studies manipulating key proteins in combination with different dDNA types can go some way to explaining the variable effects of small molecules on PGE rates. However, a more complete understanding of the precise roles of these proteins in faithful repair mechanisms is still required. For example, Lamas-Toranzo and colleagues suggested that RS-1 can stimulate other Rad51-like proteins, and this could explain how combining HDR/Rad51-promoting RS-1 with an SSTR-related ssODN donor can still increase the rate of PGE (Lamas-Toranzo et al., 2020).

Outcomes of small-molecule treatments could potentially also be affected by the chosen model system or Cas variant. For example, Cas12 produces staggered DNA ends, whereas Cas9 can generate blunt and staggered ends (Riesenberg and Maricic, 2018; Shou et al., 2018). This may alter how DSB repair proteins and the chemicals that modulate them take effect. However, the reasons why small molecules may not reproduce the same results in all model organisms, cell types or with different dDNA remain unclear. Typically, studies in which panels of compounds have been assessed for their effects on PGE identified at least one chemical that increased PGE efficiency, but different studies often identified different beneficial chemicals (Aksoy et al., 2019; Zhang et al., 2018; Riesenberg and Maricic, 2018). These discrepancies could be caused by the aforementioned differences in dDNA and cell type or by other minor protocol differences, which add additional variables and make direct comparisons difficult. Despite the lack of consensus, these studies do suggest that small molecules can effectively increase PGE, even if the optimal choice must be carefully selected based on the model system and dDNA. The range of chemicals, and their effects on PGE, are broad. We have summarised these in Table 1 and they have been covered in other recent reviews (Bischoff et al., 2020; Yeh et al., 2019).

## Cell cycle arrest and optimisation of DSB resolution timing

One of the reasons why PGE may be less efficient than NHEJ-mediated indels is the limited cell cycle window in which faithful DNA repair can occur. Therefore, researchers have sought to synchronise the cell cycle phase for timed delivery of Cas proteins and donors to increase PGE rates (Fig. 2B). The microtubule polymerisation inhibitors nocodazole and ABT-751 have been used to arrest human embryonic cell lines, induced pluripotent stem cells, neural progenitor cells and HEK293T cells in the S/G2 phases of the cell cycle, resulting in a significant increase in both HDR and NHEJ events (Yang et al., 2016; Lin et al., 2014). As an alternative approach, researchers have generated a Cas9-Geminin fusion. The presence of Geminin targets this fusion for ubiquitin-mediated degradation in the M and G1 phases of the cell cycle. Expressing this fusion in HEK293T cells limits Cas9, and therefore DSB formation, to the HDR/SSTR-active cell cycle stages, leading to increases in PGE rates (Gutschner et al., 2016).

These techniques, however, may be difficult to translate into *in vivo* model generation, where fertilised embryos may be harvested at different time points. In practice, many embryos need to be microinjected in sequence. Holding embryo development at the one-cell stage or in the cell cycle phases that permit HDR/SSTR, therefore, is desirable. This is especially important in organisms with rapid post-fertilisation cell cycles, such as zebrafish (Kimmel et al., 1995). A simple protocol modification to incubate zebrafish embryos on ice before microinjection of the gene-editing components showed a non-significant increase in PGE in ice-cooled compared to room temperature embryos. However, when the addition of the small molecules NU7441 and RS-1 was combined with ice incubation, this resulted in a significant 1.5- to 2-fold increase in PGE over the use of the small molecules alone (Aksoy et al., 2019). This suggests that, in zebrafish embryos at least, a straightforward protocol adaptation to slow the cell cycle, when combined with alteration of DNA repair mechanisms via small molecules, can produce significant improvements to PGE efficiency.

## The importance of gRNA design

gRNAs enable the ‘homing mechanism’ of Cas endonucleases and are thus a core part of the CRISPR system that have also been targeted for optimisation. However, some gRNAs direct Cas to the target site more efficiently than others, inducing DSBs more frequently. The reasons for this variable efficiency are incompletely understood, but sequence preferences that allow the complex to locate the target loci are thought to be crucial (Moreb and Lynch, 2021). In addition, poor gRNA specificity can lead to off-target effects (Wong et al., 2015; Manghwar et al., 2020; Schaefer et al., 2017; Varshney et al., 2015; Akcakaya et al., 2018). Furthermore, the requirement of a precise PAM site means that the positioning options of a gRNA and therefore the Cas-induced break are finite (Sternberg et al., 2014). To assist optimal gRNA design, researchers have designed a multitude of computational tools based on large datasets of on-target and off-target efficiencies (Heigwer et al., 2014; Montague et al., 2014; Moreno-Mateos et al., 2015) that provide metrics by which users can judge candidate gRNA sequences. The on-target score predicts the rate at which a given gRNA should induce cutting at the target locus, and the off-target score provides the predicted Cas endonuclease activity at unintended loci. These design tools have been comprehensively described and compared elsewhere (Chuai et al., 2017). It is clear, however, that the *in silico*-designed gRNAs still require *in vivo* and *in vitro* validation, and the efficacy of design tools across different cell types has been questioned (Chuai et al., 2017). In zebrafish, it has been found that two gRNA design tools underestimated the indel generation rate of gRNAs by ∼20% (Uribe-Salazar et al., 2022). In our own experience in zebrafish using Cas9 RNPs, even gRNAs with predicted low on-target scores (<40) can achieve cutting of >80% (as judged by indel rates in the absence of dDNA). Many tools, however, are based on algorithms trained on gRNA screens in mouse and human cells (Doench et al., 2014), and so may be more accurate in mammalian systems. The development of species-specific gRNA design tools, especially with in-built functionality for PGE, could be a valuable contribution to the field.

Although off-target edits do not directly affect PGE efficiency, they have the potential to reduce the validity of the modified cell or organism through unwanted indels in other genes (Schaefer et al., 2017). When attempting to achieve PGE, the distance from the DSB to the intended mutation site is also important. Cas9 induces a cut 3 bp upstream of the PAM recognition site (Chen et al., 2014), and mutation site distances of >15 bp from this cut result in suboptimal PGE efficiency, with an increase in distance having a more pronounced negative impact (O'Brien et al., 2019; Schubert et al., 2021; Wang et al., 2016). However, these studies also showed that the cutting efficiency of the gRNA still has a greater magnitude of effect on PGE efficiency than distance between cut and mutation site. Experimentally, gRNAs with higher cutting efficiencies can outperform gRNAs with lesser cutting efficiencies that cut closer to the edit site (Schubert et al., 2021). When choosing gRNAs, it is advisable to aim for those with a PAM site as close as possible to the site of the desired edit, with a high on-target score and low off-target score to avoid unwanted mutations.

## Rational design of donor sequences and limitations

As PGE mediated by HDR and SSTR requires a donor template, several studies have compared different dDNA designs and their effects on PGE efficiency, again often reaching different conclusions (Zhang et al., 2018; Bai et al., 2020). One group investigated whether ssODNs with either symmetrical or asymmetrical arms of homology (with relation to the length of the sequence either side of the cut site) could improve PGE efficiency. Indeed, they achieved HDR rates of up to 60% in HEK293 cells by using asymmetric ssODNs with complementarity to the non-target DNA strand (which is not bound by the gRNA) (Richardson et al., 2016). The use of ssODNs with non-target DNA strand specificity has also been validated by other researchers (Janssen et al., 2019). Multiple groups have investigated the length of dDNA homology arms, often reaching different conclusions (Table 1). According to these studies, the optimal length of ssODN homology arms varies between 30 bp and 60 bp (Okamoto et al., 2019; Wang et al., 2016; Schubert et al., 2021). However, the inability to generate ssODNs longer than 200 bp has previously been a limiting factor, both to the length of homology arms and for the length of the exogenous insert, e.g. limiting the ability to insert fluorophore tags at a specific site. Subsequent research has allowed the development of lssODNs that are up to ∼2.0 kb in length (Miura et al., 2018), enabling the design of 300 bp homology arms and the insertion of up to 200 bp of exogenous sequence (Bai et al., 2020; Ranawakage et al., 2021).

An alternative approach to lssODNs is to deliver donor templates as plasmids, which are linearised in the cell by Cas9 to produce dsDNA donors *in situ* (Wierson et al., 2020; Zhang et al., 2017a). This approach has achieved high PGE efficiencies in *Drosophila melanogaster* (Kanca et al., 2019) and up to 77% PGE in zebrafish (Hisano et al., 2015). However, there is disagreement over the optimal length of homology arms in the linearised dDNA, with one study suggesting very short, 24-48 bp homology arms for PGE in zebrafish and in porcine and human cell lines (Wierson et al., 2020), and other studies suggesting relatively long homology arms of 600 bp as being the most efficient (Zhang et al., 2017a).

The introduction of silent mutations within dDNA sequences should also be considered. Several studies have identified that a silent ‘blocking’ mutation within the PAM site of the linearised dDNA, which should prevent re-cutting of the edited locus by residual Cas, increases PGE rates (Okamoto et al., 2019; Paquet et al., 2016; Schubert et al., 2021). To aid subsequent screening, silent mutations that add or remove restriction enzyme sites (Paulsen et al., 2017; Schubert et al., 2021) can identify edited loci without the need to resort to sequencing.

Aside from specific dDNA design, dDNA availability is also a key aspect of optimising PGE. Because dDNA is physically required at the site of the DSB to act as an HDR template, increasing dDNA concentration is a logical approach to maximise its availability. However, high concentrations of ssODNs (Kagita et al., 2021; Nakanishi et al., 2015) and dsDNA can be cytotoxic (Luecke et al., 2017; Nguyen et al., 2020). An alternative approach is to fuse dDNA directly to the Cas endonuclease (Savic et al., 2018) or the gRNA (Lee et al., 2017), thereby optimising the spatial and temporal colocalisation of the template to the DSB site and increasing PGE rates, which has been effective in cell culture.

## Other considerations to optimise PGE via modulation of DSB resolution

As we discussed above, manipulation of key proteins within the DSB repair pathways, either via chemical modulation, overexpression or knockdown, can have robust and reproducible success in increasing PGE rates. In theory, there is no limit to the number of DNA repair proteins that could be simultaneously targeted for upregulation or downregulation. Indeed, combinations of up to seven chemicals to alter DSB repair protein function in cell culture have been investigated (Riesenberg and Maricic, 2018). The resulting degree of change in PGE rates depended on cell type, and whether Cas9, nCas9 or Cas12a was used, with the DNA-PKcs inhibitor NU7026 providing the bulk of PGE improvement when using standard Cas9. However, in other cases, the use of only two agents, one downregulating NHEJ and one upregulating HDR/SSTR, have successfully increased PGE rates in cell culture (Paulsen et al., 2017) and in zebrafish (Zhang et al., 2018). It remains to be seen whether the increases in PGE rates that can be achieved by directly altering DSB repair protein function have an upper limit.

Overexpression of key faithful repair components or knockdown of NHEJ proteins aim to increase the abundance of proteins interacting with a DSB leading to PGE. It is also important to consider the rates of NHEJ alongside those of PGE. *In vivo*, where genome editing affects a whole organism, the resulting animals often carry both NHEJ and PGE events (Zhang et al., 2018; Cui et al., 2018). As such, it can be useful to compare rates of PGE relative to indel formation as each animal will likely carry both types of genetic edit. The absolute degree of somatic PGE is also relevant for its germline transmission (Aksoy et al., 2019), which is important when the goal is to generate a stable line (see Box 1). The use of nCas9 variants and other methods that avoid DSBs (Anzalone et al., 2019; Gaudelli et al., 2017) sidestep the possibility of indel formation via NHEJ, thereby effectively increasing PGE rates.

The physical properties of the genomic landscape targeted for PGE are also worth considering. In HEK293 cells, targeting physically restrained heterochromatin rather than open euchromatin improved the PGE-to-NHEJ ratio (Janssen et al., 2019). In this study, HDR rates using dsDNA donors varied little between heterochromatin and euchromatin, whereas using ssODNs increased absolute PGE rates at euchromatin over heterochromatin. However, in both experiments, the authors observed higher rates of NHEJ when euchromatin was targeted, offsetting any gains in PGE. The use of multiple histone deacetylase inhibitors, which promote an open chromatin state, has also resulted in PGE and NHEJ increases in induced pluripotent stem cells (Zhang et al., 2021). The researchers noted, however, that some of this improvement was due to increased Cas9 and gRNA expression from their plasmid. However, at least in zebrafish, Cas9 reportedly lacks a preference for binding to heterochromatin or euchromatin (Moreno-Mateos et al., 2015). Gene locus- and cell-type-dependent variation in PGE efficiency has been noted in many studies (Bosch et al., 2020; Miyaoka et al., 2016; Remy et al., 2017; Petri et al., 2021), which may be due to chromatin structure or gRNA design limitations for a specific locus, although the factors controlling this variation are incompletely understood and warrant further investigation.

The best approach for optimising CRISPR-based PGE varies by model system and by the kind of edits that are desired. A consensus on optimal approaches has yet to be reached in the field, but the recent advancements described here provide some general advice. For small insertions and substitutions, the use of ssODNs complementary to the non-target strand, with chemical or protein modulation, may yield good PGE rates, whereas PGE of larger inserts may require the use of plasmid or lssODN donors, each with their own complement of chemical or protein modulation. When choosing targets for PGE, it may also be worth considering targeting multiple genes of interest, as some may be more amenable to editing.

## BE and PE: the next generation

**Fig. 3.**
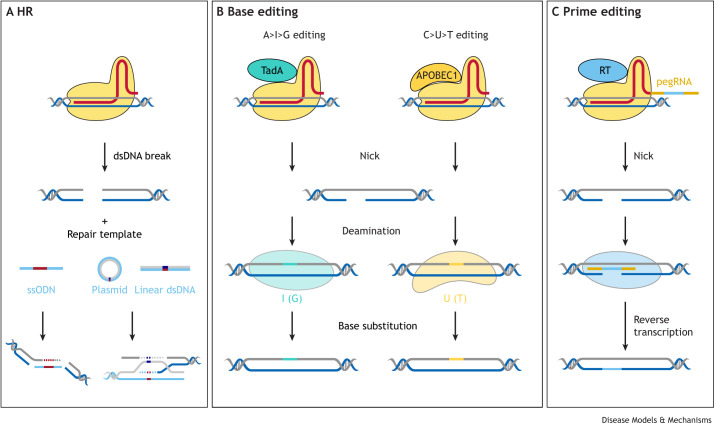
**Comparison of next-generation PGE methods.** (A) HR-based PGE, which encompasses HDR and SSTR, requires a Cas nuclease that induces a DSB in the endogenous genomic DNA, an exogenous donor DNA template that includes the desired edit, and resolution by an endogenous DNA repair pathway. (B) Base editing typically uses Cas9 nickase (nCas9), which only cuts one DNA strand (nick), fused to a deaminase. The deaminase converts adenosine to guanine or cytosine to thymine. No exogenous repair template is required. (C) Prime editing uses nCas9 fused to a reverse transcriptase and an extended gRNA (pegRNA) that acts as a repair template for the reverse transcriptase. A cut on only one DNA strand is necessary. A, adenosine; C, cytosine; dsDNA, double-stranded DNA; G, guanine; HR, homologous recombination; I, inosine; pegRNA, prime editing guide RNA; RT, reverse transcriptase; ssODN, single-stranded oligodeoxynucleotide; T, thymine; U, uracil.

Employing Cas nucleases to form DSBs followed by endogenous DNA repair is only one approach to achieve PGE. Newer techniques that rely on fusing Cas to effector enzymes, chiefly reverse transcriptase and cytidine deaminase (Fig. 3), effectively achieve PGE in cell culture and *in vivo* in zebrafish and mice (Anzalone et al., 2019; Böck et al., 2022; Gaudelli et al., 2017; Kim et al., 2017a; Koblan et al., 2018; Komor et al., 2016; Liu et al., 2020; Petri et al., 2021; Rosello et al., 2022; Sasaguri et al., 2018; Walton et al., 2020; Xu et al., 2020; Zhang et al., 2017b) (summarised in Table 2). Typically, these approaches utilise nCas9 variants that cause single-stranded DNA nicks, avoiding the bulk of deleterious NHEJ events that can result from DSB repair (Fig. 3).

**Table 2.**
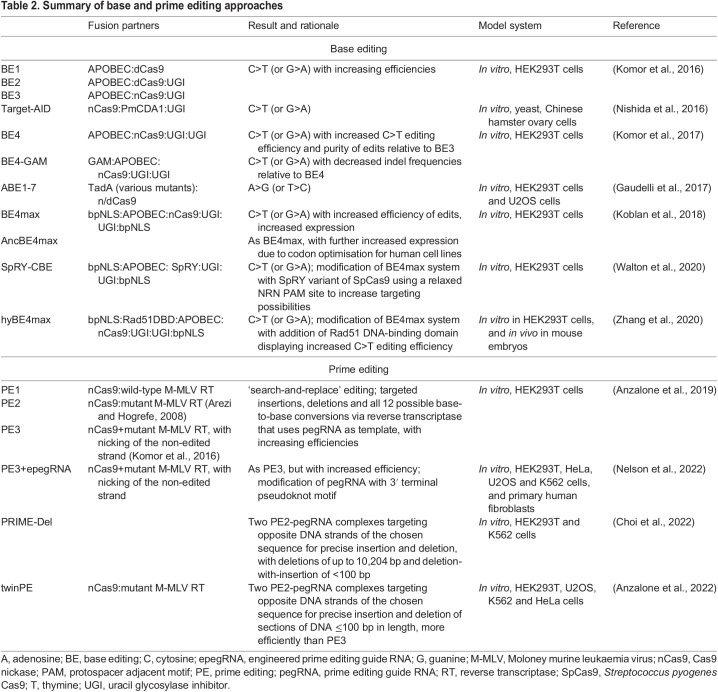
Summary of base and prime editing approaches

BE was the first to step away from manipulating endogenous DSB repair pathways. The original iteration of this system used dCas9 fused to the deaminase APOBEC1 (Harris et al., 2002; Petersen-Mahrt et al., 2002), which can ultimately convert any cytidine residues within a five-nucleotide PAM-upstream activity window to thymine (Komor et al., 2016). The more recently developed adenosine base editors are constructed with nCas9 fused to an evolved transfer RNA adenosine deaminase, which expands the BE toolbox for A>G conversion (Gaudelli et al., 2017). Researchers have continued to improve this system, increasing the base conversion edit efficiency, narrowing the deaminase activity window to increase targeting specificity, and adding PAM-less Cas9 nucleases to increase the targeting scope of the system (Gaudelli et al., 2017; Koblan et al., 2018; Komor et al., 2016; Walton et al., 2020) (Table 2). These improvements, culminating in the development of BE4, achieve a maximum editing rate of almost 70% base conversion in human cell culture (Komor et al., 2017), but these rates vary depending on the application and organism (Table 2). They range from low efficiencies of 1.3% in wheat (Zong et al., 2017) and 9-28% in zebrafish (Zhang et al., 2017b) to high efficiencies of 96% in yeast (Nishida et al., 2016), with varied levels of efficiency reported for mammalian *in vitro* (∼30-70%) and *in vivo* (44-57%) systems (Komor et al., 2017; Kim et al., 2017a). Recent attempts to apply BE to zebrafish have yielded better results, with some experiments generating up to 50% of larvae showing high rates of BE homozygosity, and up to 100% bearing some degree of BE (Rosello et al., 2022; Liang et al., 2022). Importantly, concurrent indel formation, which can be an issue in DSB pathway-based PGE methods (Zhang et al., 2018; Cui et al., 2018), is typically very low in most applications of BE and ranges between 2% and 10% (Anzalone et al., 2019; Zhang et al., 2017b), giving the technique an advantage.

The purpose of using BE is often to replicate diseases caused by single-base changes in model organisms, as has been done so successfully in zebrafish (Rosello et al., 2021), mice (Sasaguri et al., 2018) and *Drosophila* (Marr and Potter, 2021). However, the somewhat indiscriminate activity window of the deaminase is a key drawback, as it introduces a downstream screening issue, which reduces the potentially higher efficiency of the technology due to fewer indels. If a desired edit is a single-base conversion within an area that contains multiple target residues, then it is likely that BE will generate unwanted mutations. Another limitation of BE is its inability to introduce larger targeted insertions or multiple different base pair changes simultaneously. BE does have advantages over methods that rely on DSBs, but it is not yet a silver bullet for the targeted generation of single-base changes, although continued improvements may ameliorate these issues.

PE (Anzalone et al., 2019) is a still more recent advancement and again uses a nCas9 protein as a targeting chassis for an enzyme. However, in place of a deaminase, nCas9 is fused with a reverse transcriptase, such as the Moloney murine leukaemia virus (M-MLV) reverse transcriptase and bound to an extended gRNA that acts as a reverse transcription template, referred to as prime editing gRNA (pegRNA). PE has wider applicability than BE, as it can insert up to 44 bp of exogenous sequence and delete up to 80 bp (Lin et al., 2020; Anzalone et al., 2019), and it can generate any combination of base substitutions within those boundaries (Liu et al., 2020; Xu et al., 2020). Initial efforts to construct, optimise and apply PE in HEK293T cells resulted in the generation of three prime editor versions, PE1-3 (Table 2). PE3 achieved an average point mutation efficiency of 36±8.7%, with an average concurrent indel formation of 8.6±2.0% (Anzalone et al., 2019), and is more efficient than previous PE versions at generating larger precise deletions of varied sizes, from 5 bp to 80 bp, displaying editing efficiencies of 52-78% with average indel formation rates of 11±4.8% (Anzalone et al., 2019).

PE has been successfully applied to *in vivo* models, although, as is often the case with PGE, *in vivo* efficiency is generally much lower than that in cell culture. In zebrafish, PE3 did not always generate more efficient PGE than its predecessor PE2, although altering microinjection and embryo incubation temperature improved overall PE efficiency (Petri et al., 2021). The authors achieved average somatic PGE rates of up to 3.33% and 6.53% for two separate loci. From 14 edited F0 fish, only one passed PGEs onto the F1 generation at a rate of 8.3% (Petri et al., 2021). A separate study of PE in mice also identified discrepancies between the *in vivo* efficiencies of PE2 and PE3 compared to their efficiencies in mammalian cell culture (Aida et al., 2020 preprint). Here, PGE occurred in 44-75% of blastocysts, but the frequency of those PGEs accounted for only 1.1-18.5% of total embryo genomic DNA. The degree of editing within an embryo is an important factor, and one which complicates the process of generating PGEs *in vivo*. Higher rates of mosaicism (differences in the genetic sequence harboured by different cells within an organism) cause complications when trying to establish future generations that are genetically homogenous carriers of the desired edit. In addition, low rates of PGE within a single organism mean that few of the organism's offspring will likely contain the desired edit. Both outcomes cause more downstream work for researchers. An ideal technique will achieve a high number of individual embryos that bear any degree of editing, and each of those embryos should have a high percentage of their cells that bear the PGE. In another study, adeno-associated virus-mediated PE of newborn mice achieved G>C conversion with an efficiency of 14.4±6.6% in primary hepatocytes isolated at 4 weeks post injection, and the authors did not detect any indels (Böck et al., 2022). A preprint by Aida et al. indicates that PE in mouse embryos resulted in 10.5-13% PGE compared to a rate of 24-36.95% PGE when using Cas9 with ssODN donors. However, they also observed a much higher rate of PE-mediated indel formation than initially reported for mammalian cell culture (Aida et al., 2020 preprint). In *Drosophila*, PE2 has also been successfully implemented to generate efficient germline transmission of specific edits, with 36% transmission of PGEs to progeny (Bosch et al., 2021).

PE is clearly a promising step forward in PGE; but, as is often the case with new technologies, there are hurdles that need to be overcome before it can become a routine and robust tool for use in varied model organisms. The generally low indel formation and versatility of precise deletion, insertion and substitution in one platform are advantages that certainly make the continued development of PE worthwhile. In summary, BE is effective, especially when off-target mutations in the activity window are acceptable or if the targeted base is the only C or A residue in the activity window. PE can also be used to target larger indels. It is currently unclear whether previously tested chemical or protein modulation can further increase BE or PE efficiencies.

## Conclusions

Using CRISPR systems to generate gene knockouts via NHEJ remains easier than PGE. However, the refinement and improvement of methods to leverage endogenous HDR and SSTR, and the development of new techniques such as BE and PE, are paving the way for routine PGE. These advances can be effectively applied to disease modelling but still require careful selection and design of the PGE components, optimisation for a specific locus or model system, and the screening of large numbers of animals or cells to isolate those that carry the desired edits. Comprehensive comparisons of technical modifications and their effect on PGE efficiency across loci and model systems would aid a methodological consensus benefitting multiple fields of research.

With continued improvement of PGE methods, however, the future of engineering precise human disease models looks bright. Increased reliability and reduction of labour time facilitates the high-throughput or more specialised use of PGE. For example, the introduction of patient-specific mutations into animal models to generate patient ‘avatars’ would allow for pre-testing of drugs and treatments. This strategy is already being used in zebrafish and *Drosophila* for cancer patients (Bangi et al., 2021; Fazio et al., 2020). Additionally, genome-wide association studies are identifying thousands of disease-related single-nucleotide polymorphisms (Visscher et al., 2012; Yengo et al., 2022) and novel genetic pathways from gene networks (Mäkinen et al., 2014), which ultimately require validation *in vivo*. Large-scale PGE screens could be carried out to investigate these, potentially in combination, to model complex traits and assess the phenotypic impact of multiple genetic variants. Precise mutations and tagging of endogenous genes also allow for the study of how misfunctioning proteins interact.

Generating disease models to study the subtle effects of genes bearing deleterious mutations is only one application of Cas-based PGE, however. There is also huge potential for PGE to treat human disease. Recently, the CRISPR system has been used to knock out genes repressing foetal haemoglobin production to treat sickle cell anaemia in somatic cells, and additional clinical trials are underway (Kan and Doudna, 2022). Additionally, multiple clinical trials of CRISPR-edited T cells for cancer immunotherapy are underway (Lu et al., 2020; Stadtmauer et al., 2020; Kan and Doudna, 2022). Although the transition from disease modelling to treatment raises further efficacy and ethical implications, with efficient delivery, off-target screening and safety protocols all requiring careful implementation, this future application of PGE represents an exciting new use for this technology.
